# Melatonin Improves Quality of Repeated-Poor and Frozen-Thawed Embryos in Human, a Prospective Clinical Trial

**DOI:** 10.3389/fendo.2022.853999

**Published:** 2022-05-13

**Authors:** Zhongjian Bao, Guangdong Li, Rongxiang Wang, Songguo Xue, Yong Zeng, Shoulong Deng

**Affiliations:** ^1^Reproductive Center, Zaozhuang Maternal and Child Health Care Hospital, Zaozhuang, China; ^2^Beijing Key Laboratory of Animal Genetic Improvement, National Engineering Laboratory for Animal Breeding, Key Laboratory of Animal Genetics and Breeding of the Ministry of Agriculture, College of Animal Science and Technology, China Agricultural University, Beijing, China; ^3^Center for Reproductive Medicine, Shanghai East Hospital, Tongji University School of Medicine, Shanghai, China; ^4^Shenzhen Key Laboratory of Reproductive Immunology for Peri-Implantation, Shenzhen Zhongshan Institute for Reproduction and Genetics, Fertility Center, Shenzhen Zhongshan Urology Hospital, Shenzhen, China; ^5^National Health Commission of China (NHC) Key Laboratory of Human Disease Comparative Medicine, Institute of Laboratory Animal Sciences, Chinese Academy of Medical Sciences and Comparative Medicine Center, Peking Union Medical College, Beijing, China

**Keywords:** melatonin, human, embryo quality, *in vitro* culture, repeated-poor, vitrified-warmed

## Abstract

**Objective:**

In this study, two experiments were performed to assess the effect and the role of melatonin on human *in vitro* embryo quality.

**Methods:**

Experiment I: A total of 42 repeated-poor-quality-embryo patients were enrolled, with a total of 181 oocytes retrieval cycles. After IVF, for the same patient, the MT cycles group (10^-7^ M melatonin added to the culture medium; n=48) were compared with the previous non-MT cycles group (n=133), following by *in vitro* culture to blastocyst stage and embryo transfer. 31 patients were transplanted with 65 embryo transfer, including 24 MT embryo transfer, 41 non-MT embryo transfer. Cycle outcomes were compared between the two groups. Experiment II:A total of 143 supernumerary human cleavage-stage embryos (from non-repeated-poor-quality-embryo patients) vitrified on Day 3 after IVF were warmed and randomized into two groups: melatonin group (10^-7^ M melatonin added to the culture medium; n=71) and control group (n=72), and then cultured for 72 h. Rate of blastocyst and high-quality blastocyst, reactive oxygen species (ROS) levels of culture media as well as embryonic *GPX1*, *CAT*, *Mn-SOD*, *Cu/Zn-SOD*, *BCL-2*, *BAX* gene expression levels were analyzed.

**Results:**

Experiment I: Results showed that the rate of Day 3 high-quality embryos (29.6% *vs.*19.5%) in the MT cycles group was significantly higher than that in the non-MT cycles group (*P*<0.05). The rate of available blastocysts (17.1% *vs.*12.7%) and clinical pregnancy rate (25.0% *vs.*17.1%) were in tendency higher in the group treated with melatonin (*P*>0.05). Experiment II:Results showed that the blastocyst rates in the melatonin administered group were significantly higher than in control group (42.25% *vs.*26.38%, *P*<0.05). There were no significant differences in high-quality blastocyst rates. In addition, quantitative PCR showed that the expression of *CAT* was significantly upregulated by melatonin treatment (*P*<0.05), while there were no significant differences in the expression of *GPX1*, *Mn-SOD*, *Cu/Zn-SOD*, *BAX* and *BCL-2* gene as well as the levels of ROS.

**Conclusion:**

These data showed that melatonin supplement in the culture medium will improve Day 3 high-quality embryos rate of repeated-poor-quality-embryo patients and improve blastocyst rate of vitrified-warmed cleavage-stage embryos, suggesting that melatonin intervention may provide a potential rescue strategy for IVF failures.

**Clinical Trial Registration:**

identifier [ChiCTR2200059773].

## Introduction

Despite significant advances in assisted reproductive technology (ART) over the last several decades, the great majority of women who undergo fail to conceive after their first IVF cycle. A lot of patients experience repeated IVF-embryo transfer failures (1), and their following IVF cycles have a lower success rate than the general success rate. There was a specific subgroup of patients, showing a recurrent poor-quality embryo morphology phenotype and repeatedly IVF failures (2). The potential reasons for IVF failure include embryonic quality defects, reduced endometrial receptivity, and other multifactorial reasons (3). Improving the clinical pregnancy outcome in these patients is a challenge faced by clinicians practicing reproductive medicine and sub-optimal *in vitro* culture environment is somewhat responsible for frequent failures (4). In addition to select the highest quality embryos for transfer, the number of embryos available for freezing and subsequent transfers influences the cycle’s likelihood of pregnancy. At the cleavage stage, many clinics select good-quality embryos for transfer and cryopreservation, whereas poor-quality embryos are rejected (5). Thus, improving embryo quality is essential to increase the IVF transfer success rate. Cryopreservation of embryos has been widely applied and embryo cryopreservation with limited loss in viability is essential for the success of assisted reproductive technologies. However, after cryopreservation, the implantation potential of human embryos will be diminished (6, 7). In addition, as measured either by post-warming survival in culture or by established pregnancies after embryo transfer, the survival of cryopreserved *in vitro*-produced (IVP) embryos is far below that of *in vivo*-derived embryos (8). Furthermore, cultured embryos which developed to blastocyst possess a better chance of achieving implantation. However, the blastocyst rate derived *in vitro* is still lower than those derived *in vivo* (9). Although the cause of these variances are not completely understood, it seems that the inferior culture conditions may at least partly account for this difference, because reactive oxygen species (ROS) are inevitably generated during *in vitro* embryo culture, thereby resulting in an elevated ROS production and oxidative stress (OS) (10). The consequent OS may subsequently diminish the viability of embryos and jeopardize blastocyst development *in vitro* (11). Furthermore, during post-warming culture, ROS detoxification seems to be critical for embryo ability and blastocyst development.

Melatonin (5-methoxy-N-acetyl tryptamine) is a small, amphiphilic indoleamine endocrine hormone secreted primarily by the pineal gland, it has also been detected in a wide variety of extra-pineal organs, more specifically in ovary and testis in mammals as well as in many organisms, ranging from invertebrates to diverse plants and even in microorganisms (12). Melatonin plays a key role in various important physiological functions, including regulation of circadian rhythms, as well as sleep, anti-aging, and antioxidant actions (13). Melatonin and its metabolites have been shown to act as strong direct free radical scavengers and indirect antioxidants by influencing the gene expression of antioxidant enzymes (14). Melatonin has recently gained popularity as a reliable and safe cytoprotective drug in assisted reproductive technology (ART). A growing body of evidence suggests that using melatonin in the culture medium can significantly increase sperm and oocyte quality (15). Previous studies have reported the beneficial effects of melatonin on mammalian embryonic development in mouse (16), ovine (17), bovine (18), and porcine (19), and its potential mechanism is related to its antioxidant and antiapoptotic capacities. Melatonin could enhance the blastocyst rate and mean cell number/blastocyst during post-warming culture of vitrified mouse two-cell embryos by elevating glutathione and reducing ROS production (20). In addition, melatonin promoted post-warming development of vitrified ovine embryos by reducing the level of intracellular ROS, as demonstrated by increased re-expansion and hatching rates, as well as total cell number (21). However, so far, in view of the limitation of the preciousness and small quantity of human clinical embryo samples, there are relatively few studies on the effect of melatonin on the quality of human fresh-repeated poor or frozen-thawed embryos.

We speculate that melatonin supplement in culture media may be particularly beneficial for *in vitro* culture of human embryos. In order to prove this hypothesis, two experiments were carried out. A first experiment aimed at investigating the effect of melatonin on the fresh embryos from repeated-poor-quality-embryo patients, another was to explore the effect of melatonin on the frozen-thawed embryos.

## Materials and Methods

### Experiment Design

**Figure 1:**
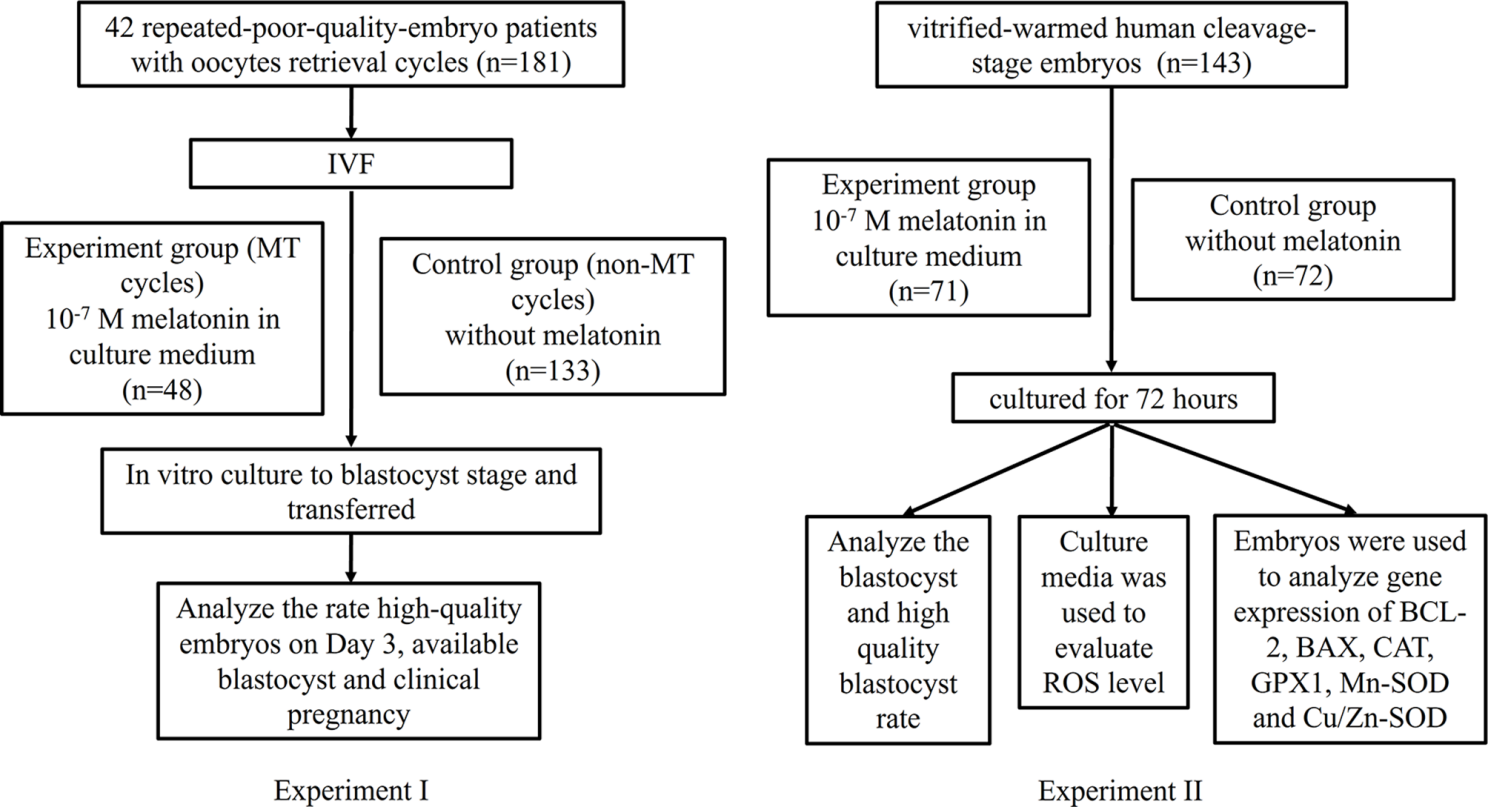
The experimental flowchart.

This study involved two experiments (experiments I, II), in which, experiment I is to investigate the effect of melatonin on the fresh embryos from repeated-poor-quality-embryo patients, experiment II is to explore the effect of melatonin on the frozen-thawed embryos from non-repeated-poor-quality-embryo patients. The experimental flowchart is shown in **Figure 1**.

In experiment I, 42 repeated-poor-quality-embryo patients with 181 oocytes retrieval cycles were enrolled. In order to exclude the influence of genetic background factors, for each patient, two groups were divided, *i.e.*, previous non-MT cycles group (control) (n=133) and MT (melatonin) cycles group (n=48). The experimental group added 10^-7^ M melatonin to the culture medium following IVF. Both groups were cultured to blastocyst stage and transferred. Rate of high-quality embryos on Day 3, available blastocyst rate, and clinical pregnancy rate were analyzed.

In experiment II, supernumerary embryos (from non-repeated-poor-quality-embryo patients) on day 3 after IVF, with a score of 1, 2 and 3 were used for vitrification-warming, and randomly cultured in culture media administered with or without 10^-7^ M melatonin for 72 h. Then, the blastocyst and high-quality blastocyst rates were evaluated. In addition, to explore its underlying mechanism, the ROS levels in culture media were quantified and the embryonic relative mRNA transcript expression levels of antioxidant genes (*GPX1*, *CAT*, *Mn-SOD* and *Cu/Zn-SOD*) and apoptosis-related genes (*BCL-2* and *BAX*) were detected.

### Ethical Statement

All embryos used in this study were donated by couples undergoing IVF treatment at Fertility Center, Shenzhen Zhongshan Urology Hospital, Shenzhen, China and Center for Reproductive Medicine, Shanghai East Hospital, Tongji University School of Medicine, Shanghai, China. For the use of their vitrified embryos, all couples signed a written informed permission form. The study was approved by Investigation and Ethics Committee of Shenzhen Zhongshan Urology Hospital.

### Patients

All patients in the *in vitro* fertilization (IVF) department between November 2017 and August 2021 who had a history of unsuccessful IVF cycles due to repeated poor embryo quality (the rate of high-quality embryos on Day 3 is less than 40%) were considered eligible for the study. A total of 42 patient with 181 oocytes retrieval cycles, were included in this study. The MT (melatonin supplementation in the culture medium) cycles group (n=48) were compared with non-MT cycles group (n=133). Basic characteristics of these patients were presented in **Table 1**. After IVF, the experimental group immediately added 10^-7^ M melatonin to the culture medium, and the control group was cultured routinely, both were cultured to blastocyst stage and transferred. Rate of high-quality embryos on Day 3, available blastocyst and clinical pregnancy were analyzed statistically. All concerned couples of this test group were informed about the study and a written consent was obtained. To confirm that there was no bias in patients-selection, statistics for the variables of age and seminal quality were evaluated.

**Table 1:** Basic characteristics of 42 patients with primarily poor quality embryos in repeated IVF cycles (n = 181).

Parameter		Values
Age (years) mean ± SD (range)		36.3 ± 5.5 (26-48)
Body mass index (kg/m2) mean ± SD (range)		22.3 ± 3.15 (18.5-32)
Infertility duration (year) mean ± SD (range)		4.1 ± 2.6 (0.5-12)
Basal FSH (IU/L) mean ± SD (range)		9.3 ± 5.4 (0.6-30.1)
Antral follicle counts in follicular phase mean ± SD (range)		7.2 ± 6.0 (1-20)
Primary infertility n (%, range in years)		17/42 (40.5)
Secondary infertility n (%, range in years)		25/42 (59.5)
Previous IVF failure n (%)	0	40/181 (22.1)
	1–2	64/181 (35.4)
	≥3	77/181 (42.5)

### Chemicals

All media were purchased from SAGE Company (SAGE, CA, USA) unless otherwise stated.

### Ovulation Induction

Ovarian stimulation was performed with the use of 300 IU recombinant FSH (Gonal-F, Merck Serono, Switzerland) and human menopausal gonadotrophin (Menotrophins for Injection, Livzon, China) after pituitary suppression by GnRHa (triptorelin acetate injection, Tiantaishan Pharmaceuticals, China) in the late luteal phase of the previous menstrual cycle. When two or more prominent follicles attained 18 mm in diameter, 6500-10000 IU human chorionic gonadotrophin (HCG, Chorionic Gonadotrophin for Injection, Livzon) was given. Oocytes were obtained by vaginal ultrasound-guided follicular aspiration 36 hours after HCG injection.

### IVF Procedures

Retrieved oocytes were collected in a G-IVF-PLUS medium (Vitro-life, Göteborg, Sweden) that was pre-equilibrated at 37°C, 6% CO_2_, 5% O_2_, 89% N_2_ in a humidified incubator. Approximately 4 h after follicular aspiration, oocytes were inseminated with 5000-10000 motile spermatozoa in G-IVF-PLUS medium. The next morning, around 19 h post-insemination, the oocytes were checked for the presence of two pronuclei (2PN), and the IVF oocytes were transferred to a pre-equilibrated GI PLUS version5 culture medium (Vitrolife). The fertilized oocytes were cultured in the culture medium (Quinn’s Advantage Cleavage Medium) containing 10% serum protein substitute (SPS) and were transferred to blastocyst medium (Quinn’s Advantage Blastocyst Medium) on day 3.

### Embryo Scoring

On the morning of day 3 (oocytes retrieval day was defined as day 0), the number of blastomeres, rate of fragmentation, size and shape of blastomeres were recorded to determine embryo quality, according to ASEBIR grading systems (22). Each embryo received a score of 1 and 2 (high quality), 3 (fair quality), 4 and 5 (poor quality). Gardner and Schoolcraft scoring system (23) was adopted to assess quality of blastocyst. First, blastocysts were ranked from 1 to 6 in terms of blastocoele diameter and expansion state. Then, based on the cell number and junction, the inner cell mass and trophectoderm were graded from A to C. The quality of blastocysts was classified as high (AA, AB, BA, and BB), fair (AC, CA, BC, and CB), or poor (CC).

### Vitrification

Embryos with a score of 1, 2 and 3 were used for direct embryo transfer and supernumerary embryos with scores of 1, 2 and 3 were vitrified for later use according to the method developed by Kuwayama (24). using the Cryotop system (Kitazato Supply, Fujinomiya, Japan) and a SAGE Vitrification Kit, with certain adjustments, according to the manufacturer’s specifications. The embryos were rated by three embryologists, who then determined which embryos to transfer or vitrify. Internally and externally, the embryo score system is validated twice a year, in accordance with the Cummins criteria (25). All of the embryos used in this investigation had been cryo-preserved for at least two years before being used.

### Embryos Warming

The warming protocol was carried out using the SAGE Vitrification Warming Kit (SAGE Trumbulll, IN, USA) according to the manufacturer’s instructions. The vitrified human cleavage-stage embryos were warmed and transferred into equilibrated Quinn’s Advantage Blastocyst Medium containing 10% (v/v) human serum albumin. A total of 143 vitrified-warmed human cleavage-stage embryos were then randomly assigned to two groups: melatonin group (10^-7^ M, n=71, according to our unpublished results that 10^-7^ M melatonin could promote *in vitro* human blastocyst development) and control group (without melatonin, n=72) based on embryo morphology/survival rate and cultured for 72 hours. The blastocyst and high-quality blastocyst rate were evaluated. In addition, embryo scores of the 143 vitrified-warmed human cleavage-stage embryos were showed in **Table 2**, there were no differences between the two groups.

**Table 2:** Embryo scores of the 143 vitrified-warmed human cleavage-stage embryos.

Groups	No. of vitrified-warmed human cleavage-stage embryos		
	No. of embryos scoring 1	No. of embryos scoring 2	No. of embryos scoring 3
Control	19	38	15
Melatonin	20	37	14

### Measurement of ROS in Culture Media

The ROS levels in culture media were evaluated by chemiluminescence assay (26) using 5-amino-2,3-dihydro-1,4- phthalazinedione (luminal; Sigma, St. Louis, MO, USA) as a probe in a luminometer (Synergy H1 Hybrid Reader; Biotek, USA). Each sample was scanned at 37°C for 15 minutes by the luminometer. ROS values were expressed as relative light units (RLUs). All the samples were measured in duplicate.

### RNA Extraction and Quantitative PCR

In addition, all the vitrified-warmed human embryos cultured for 72 h were collected and used to analyze the effect of melatonin on the gene expression of *BCL-2*, *BAX*, *CAT*, *GPX1*, *Mn-SOD* and *Cu/Zn-SOD*. Total RNAs were extracted using Power SYBR Green Cells-to-CT Kit (Life Technologies Corporation, CA, USA) according to the manufacturer’s instructions. Briefly, all the vitrified-warmed human cleavage-stage embryos from the two groups were collected and washed with phosphate-buffered saline (PBS) respectively. RNA was transcribed to synthesize complementary DNA (cDNA) using a convenient 20×RT enzyme mix and 2×SYBR RT buffer with the following settings: 37°C (60 minutes) and then 95°C (1 minute). The cDNA was then mixed with the 2×SYBR Green PCR master mix and primers for genes *BCL-2*, *BAX*, *CAT*, *GPX1*, *Mn-SOD*, *Cu/Zn-SOD* and *ACTB* (**Table 3**) in individual PCR reactions and real-time PCR was performed using a Applied Biosystems 7500 Real-Time PCR System with the following settings: 95°C (10 minutes), then 35 cycles of 95°C (15 seconds) and 60°C (1 minute). Three replicates were performed, and the expression levels of target genes were normalized to the expression level of *ACTB*.

**Table 3:** Primers used in this study.

Gene	Primer sequence (5’–3’)	Tm (°C)	Size (bp)
*GPX1*	forward: ACGATGTTGCCTGGAACTTT reverse: GATGTCAGGCTCGATGTCAA	57.8	104
*CAT*	forward:CGTGCTGAATGAGGAACAGA reverse:AGTCAGGGTGGACCTCAGTG	57.8	119
*Mn-SOD*	forward:GCTCATGCTTGAGACCCAAT reverse: CACCCGATCTCGACTGATTT	57.8	80
*Cu/Zn-SOD*	forward: GGCAAAGGTGGAAATGAAGA reverse: GGGCCTCAGACTACATCCAA	59.8	112
*BCL-2*	forward: TCTCCCTTCAGAATCTTATC reverse: AAACACCTGCTCACTCACT	63.8	83
*BAX*	forward: AAGAAGCTGAGCGAGTGT reverse: GGCGGCAATCATCCTCTG	57.9	78
*ACTB*	forward: TGGGTCAGAAGGATTCCTATGT reverse: CAGCCTGGATAGCAACGTACA	58.2	276

### Statistical Analysis

Statistical analysis was carried out using the Statistical Package for Social Sciences version 15.0 (SPSS, Chicago, IL, USA). Data were presented as mean ± SD. All variables were tested for normal distribution with Kolmogorov-Smirnov test, histogram, and P-P plots. Variables were compared with either independent samples t-test or Mann-Whitney U test depending on the normality of the data. All categorical variables were compared with Pearson Chi-square and Fisher’s exact tests. A p value<0.05 was considered as statistically significant.

## Results

### Melatonin Improves Day 3 Embryo Quality in Patients With Repeated Poor Embryo Quality

42 repeated-poor-embryo-quality patients were selected, with 181 oocytes retrieval cycles, comprising 133 non-MT cycles and 48 MT cycles. A total of 31 patients were transplanted with 65 embryo transfer, including 24 MT embryo transfer, 41 non-MT embryo transfer. As showed in **Table 4**, the rate of Day 3 high-quality embryos (29.6% *vs.*19.5%) in the MT cycles group was significantly higher than that in the non-MT cycles group (*P*<0.05). There was no statistically significant difference in the rate of available blastocysts (17.1% *vs.*12.7%) and the clinical pregnancy rate (25.0% *vs.*17.1%) between the MT cycles group and the non-MT cycles group (*P*>0.05).

**Table 4:** Results of repeated-poor-quality-embryo culture treated with MT or not.

/	Non-MT cycles group (Control)	MT cycles group	*P* value
Cycles (n)	133	48	/
Day 3 high-quality embryos rate (%)	19.5 (86/442)	29.6 (40/135)	0.0123
Available blastocyst rate (%)	12.7 (38/300)	17.1 (14/82)	0.3025
Clinical pregnancy rate (%)	17.1 (7/41)	25.0 (6/24)	0.6529

### Melatonin Improves Blastocyst Rate of Frozen-Thawed Embryos and Promotes CAT Gene Expression

**Figure 2:**
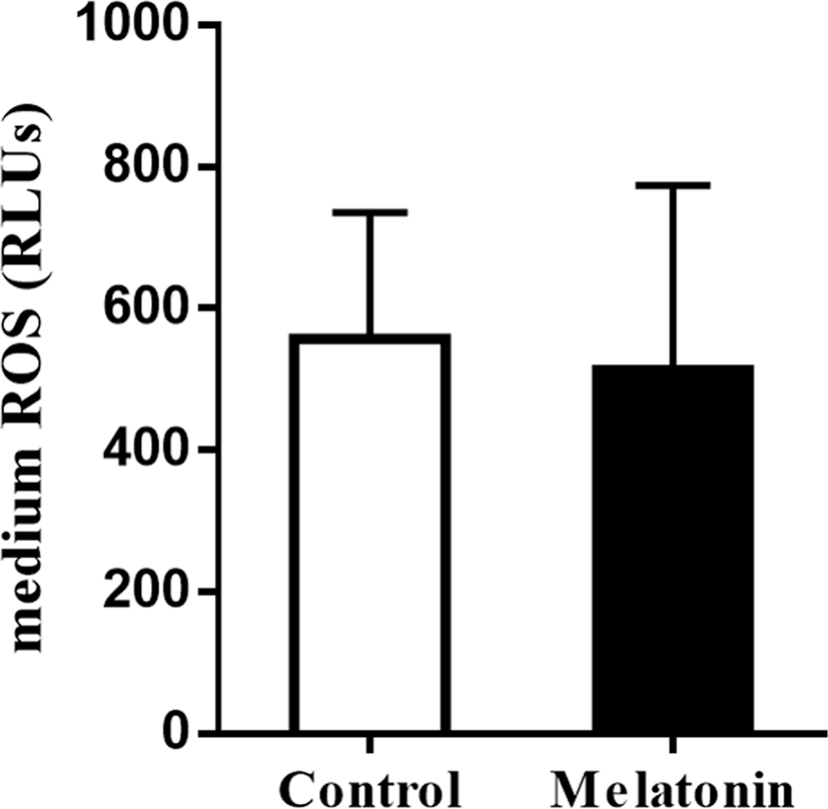
Effects of melatonin on the ROS levels of culture media culturing vitrified-warmed human cleavage-stage embryos for 72 h. Control: the ROS levels of culture media supplemented with no melatonin; Melatonin: the ROS levels of culture media supplemented with 10^-7^ M melatonin.

**Figure 3:**
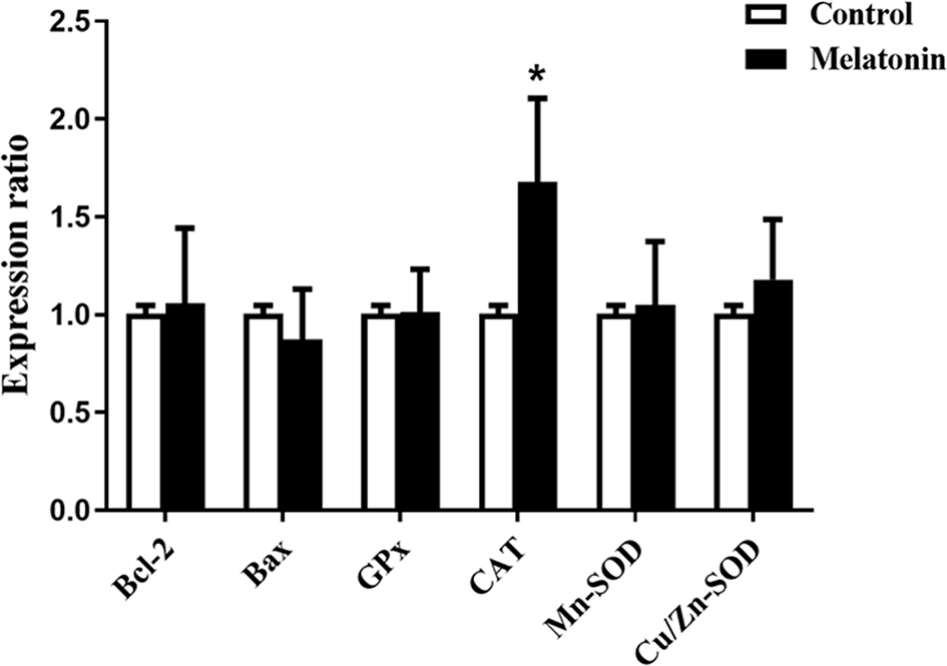
Effects of melatonin on the gene expression of BCL-2, BAX, CAT, GPX1, Mn-SOD and Cu/Zn-SOD in vitrified-warmed human cleavage-stage embryos cultured for 72 h. *P < 0.05 compared with the control group.

To explore the actions of melatonin on the *in vitro* development of vitrified-warmed human embryos, a total of 143 vitrified-warmed human cleavage-stage embryos were randomly divided into two groups. The results were shown in **Table 5**. Compared with the control group, the blastocyst rate was significantly increased by melatonin treatment (42.25% *vs.* 26.38%, *P*<0.05). Although the high-quality blastocyst rate of melatonin group was higher than that of control group, no statistically significant difference was observed between two groups (30.00% *vs.* 26.32%, *P*>0.05). To explore the effects of melatonin on ROS in culture media culturing vitrified-warmed human embryos for 72 h, the ROS levels were measured and the results were shown in **Figure 2**. Although the ROS levels in culture media of melatonin-treated group (513.33 ± 260.61) was lower than that of the control group (556.67 ± 178.47), no significant difference was found (*P*>0.05). To further investigate the underlying mechanisms of the beneficial effect of melatonin on the *in vitro* development of vitrified-warmed human cleavage-stage embryos, the expression of the apoptosis-related genes (*BAX* and *BCL-2*) and antioxidant enzymes including *Mn-SOD*, *Cu/Zn-SOD*, *GPX1* and *CAT* were examined (**Figure 3**). Melatonin administration considerably increased the expression of *CAT* when compared to the control group (1.68 ± 0.43 *vs.* 1.00 ± 0.09) (*P*<0.05). Although the expression of *GPX1*, *Mn-SOD*, *Cu/Zn-SOD* and *BCL-2* of melatonin group were higher than those of the control group and the expression of *BAX* were lower than that of the control group, no significant differences were exhibited (*P*>0.05).

**Table 5:** Effect of melatonin on the *in vitro* development of 143 vitrified-warmed human cleavage-stage embryos.

Groups	Rate of blastocysts (%)	Rate of high-quality blastocysts (%)
Control	26.38 (19/72)	26.32 (5/19)
Melatonin	42.25 (30/71)*	30.00 (9/30)

*P < 0.05 compared with the control group.

## Discussion

More and more sub-fertile couples who have been unable to conceive naturally turn to artificial reproduction techniques such as *in vitro* fertilization (IVF) to achieve successful pregnancy. IVF is the most effective treatment for infertility, with cumulative delivery rates of 45–50% per cycle (27). Repeated IVF-embryo transfer failures occurred for a variety of causes. Reduced endometrial receptivity, as well as embryonic developmental defects such as decreased embryo quality due to a poor culture environment and genetic factors, are common reasons (28). According to lots of studies, more than one-third of IVF-embryo transfer failures are due to poor embryo quality (29). When high-quality embryos are obtained, the live-birth rate increases, hence embryo quality is a crucial determining factor in IVF program success rates. IVF culture conditions are only an inferior match for the internal environment of the reproductive tract, in which, embryos are susceptible to cellular stress as a result (30). To withstand this stress and develop in such an environment, embryos must be able to synthesize required components not provided by the medium and respond to parameters imposed by the artificial surroundings.

Earlier reports have documented that melatonin not only synthesized in the neuroendocrine system but also in the reproductive system, especially in ovary and uterus. Melatonin is involved in the pathophysiology of a variety of reproductive processes. Melatonin concentrations in human preovulatory follicular fluid (FF) are higher than in plasma (31). There was a positive correlation between follicular fluid melatonin levels and *in vitro* fertilization outcomes as well as oocyte quality (32). In clinical research, approximately 3% of couples in IVF programs exhibited an unpredictable and recurrent poor-quality embryo morphology phenotype, associated with recurrent IVF failures. In our study, for these patients, the results showed that melatonin treatment significantly increased the rate of Day 3 high-quality embryos. In addition, the rate of available blastocysts and clinical pregnancy rate were in tendency higher in the group treated with melatonin, despite that the difference did not reach significant. The mechanisms that lead to poor embryo quality are numerous. Maternal oocyte cytoplasmic factors (melatonin is an essential one) were important regulators of early biological activities after fertilization, which played a pivotal role in governing early embryonic development. Numerous studies have shown that disrupting the circadian rhythm by night-time work, which results in light exposure changes (melatonin secretion levels are drastically reduced), has a deleterious impact on normal embryo development, implantation and pregnancy success, leading to an increase in infertility, menstruation irregularities, and miscarriages (33). Melatonin could significantly improve ratio of Grade I embryos in IVF patients with sleep disturbances (34). Previous reports also showed that oral melatonin supplementation significantly improved rate of good quality embryos (48.0 versus 65.6%) in women undergoing *in vitro* fertilization-embryo transfer (35). In agreement with our study, a similar result was reported by Khan et al. (36) They used 895 follicular fluid (FF) samples and demonstrated elevated levels of cell-free DNA (cfDNA) fragments (caused by oxidative damage to DNA) in infertile patients FF samples is negatively associated with melatonin concentration and related with embryo quality, which revealed that cfDNA levels and melatonin concentration could be used as a valid predictor for embryo quality on Day 3. Likewise, a systematic review suggests that melatonin treatment actually significantly increases the number of oocyte collected, maturated oocyte, good quality embryo and clinical pregnancy rate in ART cycles (37). Certainly, to better investigate the mechanism of melatonin actions on repeated poor-quality embryo, more clinical samples with homogeneity as well as in-depth molecular biology experiments should be carried out.

In the current study, we confirmed that adding melatonin to culture media significantly boosted the *in vitro* growth of vitrified-warmed human cleavage-stage embryos, as evidenced by a higher blastocyst rate. The result was similar to those previously reported in other species. Marques et al. (38) demonstrated the beneficial effects of melatonin on the development of fresh and vitrified bovine embryos including enhancement of blastocyst rate. However, there was no significant difference in high quality blastocyst rate between the melatonin and control groups in this study, which is different from those previously reported. Perhaps, in our experiment, the difference was due to that, melatonin was added to culture media during the later development stage of vitrified-warmed embryos, whereas melatonin was used in the earlier development of other species. On the other hand, we assumed that the discrepancy could also be related to melatonin concentration differences. Hao et al. (39) demonstrated that the ratio of human high-quality blastocysts was significantly higher in the groups treated with 10^−5^ M melatonin compared with other groups. Mehaisen et al. (40) found that 10^-3^ M melatonin treatment could significantly improve the quality of rabbit blastocyst derived from both fresh and vitrified embryos by lowering damages caused by oxidative stress. In addition, 10^-9^ M melatonin treatment could also improve the quality of blastocyst from vitrified human embryos by maintaining the normal ultrastructural features and the permeability of the oolemma in vitrified embryos (41). The working concentration of 10^-7^ M melatonin was chosen in this study based on our unpublished findings that this concentration may significantly improve *in vitro* human blastocyst development among various groups (0 M, 10^-9^ M, 10^-7^ M and 10^-5^ M). Moreover, high concentrations of melatonin could retard embryo development and inhibit cell division (42). To be honest, 10^-7^ M melatonin might not be the best concentration for post-warming culture of vitrified human embryos and its optimal concentration needs to be subsequently investigated.

Vitrification and warming are involved in cryopreservation of human embryos during ART. However, its major shortcoming is that it retards the early embryo development to some extent by inducing ROS production (43, 44). Therefore, excessive ROS is at least partially responsible for the developmental arrest or apoptosis in cryopreserved embryos. Under normal conditions, embryos have the capacity to defend against excessive ROS by antioxidants (GPX1, CAT, SOD, etc.), but these protective defenses may be destroyed during embryo cryopreservation (45). Thus, removal of excess ROS is especially important for post-warming culture of vitrified-warmed human embryos. As the potent antioxidant, melatonin possesses the ability to powerfully scavenge ROS. Moreover, melatonin has been shown to stimulate the growth of vitrified-warmed embryos in mice (46) and equine (47). Besides, the substantial reduction in ROS in melatonin-treated embryos has been reported in porcine (48). Thus, melatonin’s robust free radical scavenging properties may explain for its favorable effects on the development of vitrified-warmed human embryos, according to the researchers. In order to confirm this speculation, the ROS levels in culture media culturing vitrified-warmed human embryos for 72 h were quantified. In the current investigation, however, no significant changes in ROS levels in culture media was observed between the melatonin and control groups, which contradicts the above-mentioned observations in other species’ embryos. It’s possible that the differences are due to different ROS detection methods. Because in our work, the level of ROS, which included all kinds of ROS (not only the intracellular ROS produced by embryo but also those produced by environment), was measured using a chemiluminescence assay (26) with luminal as a probe, but in a previous study (44), the level of ROS (mainly the intracellular ROS), which included H_2_O_2,_ was measured using 2’,7’-dichlorofluorescein fluorescence. As a result, the ROS level found in our study may be closer to the actual level of ROS produced during embryo *in vitro* development. Furthermore, melatonin may have a pivotal role in embryonic development regulation by influencing lipid metabolism (49), MT1 receptor interaction (50), mitochondrial function, DNA methylation (51), etc., in addition to clearing ROS.

Under normal circumstances, there existed dynamic balance between ROS and antioxidants in embryo. Excessive ROS produced by embryos during *in vitro* culture not only broke the balance but also induced the apoptosis that jeopardized the development of embryos (52). Therefore, antioxidants (GPX1, CAT, SOD, etc.) responsible for scavenging excess ROS and apoptosis-related gene (BAX and BCL-2) are important for the normal embryonic development. It is well known that melatonin could upregulate GPX1, CAT and SOD expression and relieve the DNA damage in many studies (53). To further investigate the potential mechanisms by which melatonin exerts its favorable effects on the *in vitro* development of vitrified-warmed human cleavage-stage embryos, we explored the expression levels of antioxidant enzyme genes (*GPX1*, *CAT*, *Mn-SOD*, *Cu/Zn-SOD*) as well as apoptosis-related genes (*BAX* and *BCL-2*). Our findings showed that the expression of *CAT* was significantly upregulated by melatonin treatment, which was consistent with the previous report (54). However, no significant differences in the gene expression of *GPX1*, *Mn-SOD*, *Cu/Zn-SOD*, *BAX* and *BCL-2* were exhibited between melatonin-treated and control groups, which differed from that previously reported (13, 55, 56). Previous studies demonstrated that melatonin promoted bovine embryonic development by significantly upregulating the gene expression of *GPX1* and *Cu/Zn-SOD* (50). In addition, melatonin enhanced porcine SCNT blastocyst formation by increasing the expression of antiapoptotic gene *BCL-2* and decrease pro-apoptotic gene *BAX* (57). Perhaps, different species made the difference. In mouse, melatonin could also upregulate gene expression of *Sod* and *Bcl-2* but have no influence on the expression of *Gpx1* and *Bax* during *in vitro* development of embryos (45). Furthermore, in our work, melatonin was administered to culture medium during the later stages of vitrified-warmed embryos development, as opposed to earlier or whole stages in other species, which may explain the difference. Our results suggest that high levels of *CAT* may in turn boost the antioxidant capacity of human vitrified-warmed embryos, promoting blastocyst development. The limitation of our study is that the activity of CAT is not detected and the exact mechanism which results in the favorable effects of melatonin on the *in vitro* development of vitrified-warmed human embryos still requires further in-depth study.

## Conclusion

Conclusively, this study indicates that melatonin treatment favors repeated poor-quality embryos as well as frozen-warmed embryos *in vitro* development, which is potentially an efficient rescue strategy for IVF failures during ART cycles. Therefore, melatonin should be considered for patients with poor IVF and embryo quality due to its safety and ease of administration. However, large-scale trials and studies will be needed in the future to standardize the procedure before it can be used in a personalized care-program.

## DATA AVAILABILITY STATEMENT

The original contributions presented in the study are included in the article/supplementary material. Further inquiries can be directed to the corresponding authors.

## ETHICS STATEMENT

The studies involving human participants were reviewed and approved by Investigation and Ethics Committee of Shenzhen Zhongshan Urology Hospital. The patients/participants provided their written informed consent to participate in this study.

## Author Contributions

Conception or design of the work: YZ, SX, and SD; Acquisition of data, Analysis and interpretation of data: RW. Drafting and revising the article: ZB and GL. All authors have read and agreed to the published version of the manuscript.

## Funding

This work was supported by the National Natural Science Foundation of China (no. 82171691), Medical Scientific Research Foundation of Guangdong Province (no. B2010292) and Basic Research Program of Shenzhen (no. JCYJ20120829150019348 and no. JCYJ20120829150019349).

## Conflict of Interest

The authors declare that the research was conducted in the absence of any commercial or financial relationships that could be construed as a potential conflict of interest.

## Publisher’s Note

All claims expressed in this article are solely those of the authors and do not necessarily represent those of their affiliated organizations, or those of the publisher, the editors and the reviewers. Any product that may be evaluated in this article, or claim that may be made by its manufacturer, is not guaranteed or endorsed by the publisher.
